# Functional remodeling of the gut microbiome and metabolome in primary idiopathic male infertility

**DOI:** 10.1186/s12866-026-05064-x

**Published:** 2026-04-24

**Authors:** Chen Luo, Hailin Yao, Yang Xian, Tingting Yang, Xiao Xiao, Lijuan Ying, Jinyan Xu, Xuefeng Luo, Dajian Qiu, Yuan Liu, Bo Liu, Fuping Li

**Affiliations:** 1 Department of Reproductive Andrology & Sichuan Human Sperm Bank, West China Second University Hospital, Sichuan University, Chengdu, Sichuan 610041 PR China; 2 Key Laboratory of Birth Defects and Related Diseases of Women and Children, Sichuan University, Ministry of Education, Chengdu, Sichuan 610041 PR China; 3 Department of Maternal and Child Health, West China School of Public Health and West China Fourth Hospital, Sichuan University, Chengdu, Sichuan 610041 PR China

**Keywords:** Primary idiopathic male infertility, Gut microbiome, Metabolome, Shotgun metagenomics, Untargeted metabolomics

## Abstract

**Background:**

Primary idiopathic male infertility (PIMI) is a complex condition with unclear biological mechanisms. Increasing evidence indicates that gut microbiome-derived functional and metabolic alterations can influence host physiological processes, yet microbiome-associated functional changes in PIMI remain poorly characterized.

**Methods:**

In this case–control study, fecal shotgun metagenomics and untargeted liquid chromatography-tandem mass spectrometry (LC–MS/MS) metabolomics were performed in 19 men with PIMI and 12 fertile controls, alongside computer-assisted semen analysis. The study workflow integrated differential analyses, correlation analyses among key microbial species, metabolites, and clinical traits, and Random Forest modeling to derive a microbial-metabolic panel.

**Results:**

Compared with fertile controls, infertile men exhibited selective functional remodeling of gut microbial pathways and fecal metabolic profiles, accompanied by reduced sperm concentration and progressive motility and increased round cell counts. Although overall microbial diversity was broadly comparable between groups, 23 differentially abundant species and 53 altered Kyoto Encyclopedia of Genes and Genomes (KEGG) pathways were identified by metagenomic profiling. Untargeted metabolomics annotated 4,434 metabolites and identified 780 differential metabolites, with enrichment of 29 KEGG pathways. Eight key microbial species and eight key metabolites mapped to sperm- and testis-related pathways showed coordinated correlations with semen parameters. An integrated Random Forest model incorporating microbial and metabolic features demonstrated robust discrimination between infertile and fertile men, with optimal performance achieved using six top-ranked features.

**Conclusions:**

PIMI is associated with selective gut microbial functional shifts and fecal metabolic disturbances that correlate with semen quality. Multi-omics integration highlights coordinated microbiome-metabolome alterations, providing insights into host-associated microbial functional dysregulation in male infertility.

**Supplementary Information:**

The online version contains supplementary material available at 10.1186/s12866-026-05064-x.

## Introduction

Male infertility affects approximately 7% of the global male population and contributes to nearly half of all infertility cases among couples [1]. Despite significant advances in reproductive medicine, the etiology of male infertility remains unclear in nearly 30–50% of affected individuals, who are therefore classified as having idiopathic infertility [2]. Primary idiopathic male infertility, defined as infertility in men who have never achieved pregnancy and exhibit no identifiable anatomical, genetic, or endocrine abnormalities, represents a particularly challenging clinical subset. These patients often experience considerable psychological and social burdens, and the absence of identifiable causes limits both diagnostic precision and therapeutic options [3].

In recent years, increasing evidence has implicated the gut microbiome as a key regulator of host metabolism, immune activity, and endocrine function [4–6]. Alterations in gut microbial composition, commonly referred to as dysbiosis, have been associated with metabolic disorders, chronic inflammation, and disturbances in reproductive hormones [7–9]. The concept of a bidirectional communication pathway between the gut and the testes, often described as the gut-testis axis, suggests that microbial metabolites, inflammatory mediators, and endocrine signals may influence spermatogenesis and male reproductive potential [10].

Several human studies support a potential link between gut microbiome and male reproductive health. Lundy et al. demonstrated that men with infertility show distinct microbial signatures in gut, semen, and urine samples that are not observed in fertile controls [11]. Similarly, Cao et al. applied shotgun metagenomics to characterize fecal microbiome in men with non-obstructive azoospermia (NOA) and identified alterations in carbohydrate metabolism pathways together with specific bacterial taxa associated with impaired spermatogenesis [12]. Although these studies highlight the possibility that gut microbiome disturbances contribute to male infertility, research specifically targeting idiopathic forms, particularly PIMI, remains scarce. In addition, most existing studies focus on taxonomic profiling without integrating functional metagenomics and metabolomics.

Evidence from animal models further supports a mechanistic link between the gut microbiome and spermatogenesis. In antibiotic-treated mice, depletion of the gut microbiome led to testicular dysfunction and impaired spermatogenesis, accompanied by systemic metabolic disturbances and oxidative stress-related injury, highlighting a causal gut-testis crosstalk [13]. Restoration of microbial balance partially reversed these abnormalities, suggesting a causal role for dysbiosis. Jin et al. later reported that modulation of the gut microbiome ameliorated diabetes-induced spermatogenic dysfunction, largely through reshaping adenosine metabolism along the gut-testis axis, highlighting a metabolic bridge linking systemic metabolic disturbances to testicular function [14]. Additional studies have demonstrated that microbiota-derived metabolites, including short-chain fatty acids and tryptophan derivatives, can modulate host immune and endocrine signaling, thereby shaping the reproductive endocrine milieu and influencing steroidogenesis and male reproductive function [15]. These observations collectively provide plausible biological routes through which gut dysbiosis may contribute to male infertility.

At the metabolic level, idiopathic male infertility has been associated with systemic and local metabolic disturbances, including alterations in amino acid metabolism, lipid metabolism, and energy-related pathways [16]. Given that the gut microbiome is a major contributor to host metabolic output, integrating functional metagenomics with untargeted metabolomics provides a promising strategy to characterize microbe-metabolite interactions potentially involved in impaired spermatogenesis [17, 18]. However, to date, studies that comprehensively integrate these two omics layers to jointly profile the gut microbiome and fecal metabolome in men with primary idiopathic infertility remain limited [10, 11, 15].

This study aimed to investigate the taxonomic and functional features of the gut microbiome, together with fecal metabolic alterations, in men with PIMI compared with fertile controls. Using shotgun metagenomic sequencing and untargeted metabolomics, we sought to identify microbial taxa, metabolic pathways, and microbe-metabolite associations that differentiate PIMI from normal fertility. We hypothesized that men with PIMI harbor distinct microbial and metabolic signatures that reflect perturbations in the gut-testis axis and may contribute to impaired spermatogenesis despite the absence of identifiable clinical etiologies. By integrating multi-omics data, this study provides new insights into potential microbiome-related mechanisms underlying primary idiopathic male infertility and may help identify biomarkers or therapeutic targets for this condition.

## Materials and methods

### Study design and participant recruitment

**Fig. 1:**
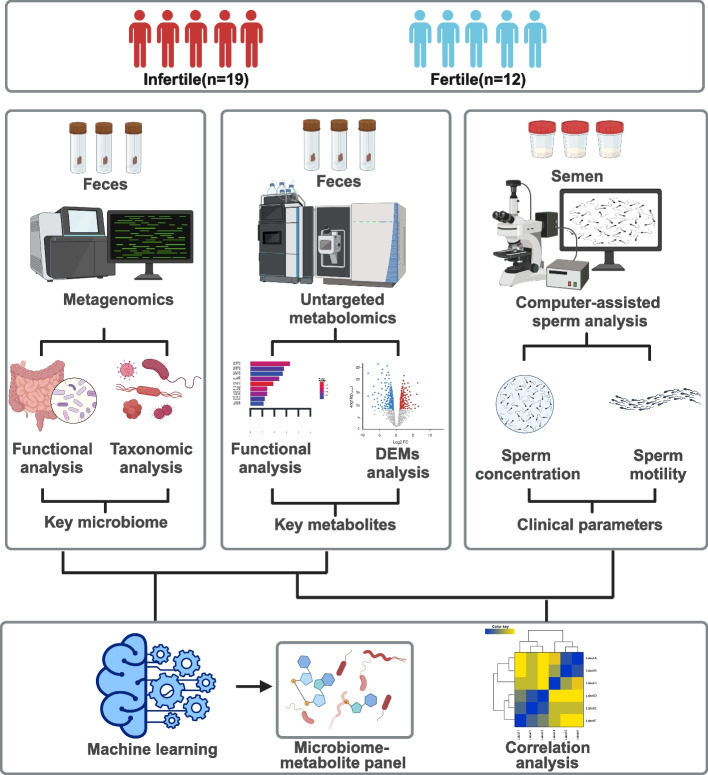
Study design and analytical workflow. Schematic overview of the study design and multi-omics analysis pipeline. Men with primary idiopathic male infertility (PIMI, *n* = 19) and fertile controls (*n* = 12) were recruited. Semen parameters were assessed, and fecal samples were collected for shotgun metagenomic sequencing and untargeted LC–MS/MS metabolomics. Differential microbial taxa and KEGG functional pathways, differential metabolites and enriched KEGG pathways, integrative correlation analyses, and Random Forest modeling were performed to identify key microbial-metabolic signatures and a candidate biomarker panel

The overall study design is summarized in Fig. 1. This case–control study aimed to compare the gut microbiome and fecal metabolome between men with primary idiopathic male infertility and fertile controls. Participants were recruited from the Department of Reproductive Medicine, West China Second Hospital of Sichuan University. Men in the PIMI group were diagnosed with primary infertility, defined as failure to achieve pregnancy after at least 12 months of unprotected intercourse. To confirm idiopathic infertility, participants underwent routine clinical, endocrine, genetic, and imaging evaluations, and those with identifiable etiologies were excluded. Exclusion criteria included: (1) varicocele (clinical grade II or above), (2) history of cryptorchidism or orchitis, (3) chromosomal abnormalities or Y-chromosome microdeletions, (4) obstructive lesions of the reproductive tract, (5) endocrine disorders affecting reproduction, (6) active genitourinary infections, (7) use of antibiotics, probiotics, or hormonal medications within the previous three months, and (8) chronic systemic illnesses known to affect fertility. Fertile controls were men with proven natural fertility (fathering a child within the previous two years) and without known reproductive, metabolic, or gastrointestinal disorders. All participants were aged 18–55 years, had no history of gastrointestinal surgery, and followed omnivorous diets. In total, 19 men with PIMI and 12 fertile controls met the inclusion criteria and were enrolled. Baseline demographic characteristics and lifestyle factors were collected following standardized procedures. This study was approved by the Medical Research Ethics Committee of West China Second Hospital of Sichuan University (approval number: 2024312). Written informed consent was obtained from all participants prior to enrollment.

### Semen collection and analysis

Semen samples were collected following 2–7 days of abstinence. Participants were instructed to obtain samples by masturbation in a dedicated collection room near the laboratory to minimize time to analysis. Sample liquefaction occurred at 37 °C for up to 60 min. Semen parameters were evaluated according to the World Health Organization (WHO) Laboratory Manual for the Examination and Processing of Human Semen (6th edition).

Routine assessments included semen volume, sperm concentration, motility, morphology, and vitality. Strict criteria were applied for morphological evaluation. Samples showing leukocytospermia or evidence of genital tract infection were excluded from the study. These semen analysis results were used to confirm infertility in the PIMI group and normal fertility status in the control group.

### Fecal sample collection and processing

Fresh fecal samples were collected from participants in sterile containers on the same day as semen examination whenever possible. Samples were immediately stored at −80 °C until further processing. All samples were randomized before sequencing and metabolomics analysis to minimize batch effects.

### Metagenomic sequencing and data analysis

Metagenomic DNA was extracted from 0.2 g of fecal material using the FastPure Stool DNA Isolation Kit (Magnetic Bead; MJYH, Shanghai, China) according to the manufacturer’s instructions. DNA concentration and purity were assessed with a NanoDrop 2000 and Synergy HTX reader, and integrity was verified on 1% agarose gels. DNA was sheared to ~ 350 bp using a Covaris M220, and paired-end libraries were prepared with the NEXTFLEX Rapid DNA-Seq Kit (Bioo Scientific, USA). Sequencing was performed on the MGISEQ-T7 platform (MGI, China).

Raw reads were filtered with fastp (v0.23.0) to remove adapters and low-quality sequences, and host reads were removed by mapping to the human reference genome using BWA (v0.7.17). High-quality reads were assembled using MEGAHIT (v1.1.2), and open reading frames (≥ 100 bp) were predicted with Prodigal (v2.6.3). A non-redundant gene catalog was generated using CD-HIT (v4.6.1) at 90% identity and coverage, and gene abundance was estimated using SOAPaligner (v2.21).

Taxonomic annotation was performed by aligning non-redundant genes against the NCBI NR database (downloaded in August 2024) using DIAMOND (v2.0.13) with an e-value threshold of 1e-5. Functional annotation of the non-redundant genes was performed against the KEGG database to assign KEGG Orthology (KO) entries, and KEGG pathway abundance was summarized based on the abundance profiles of the annotated genes. Gene abundance was normalized using the RPKM method, and taxonomic abundance profiles for downstream analyses were derived from the RPKM-normalized data and expressed as relative abundance. No additional prevalence-based filtering was applied prior to downstream taxonomic analyses. These taxonomic abundance data were used for diversity analyses and between-group comparisons. Normality was assessed using the Shapiro–Wilk test. As the taxonomic and functional abundance data were generally non-normally distributed, differential taxonomic and functional features between groups were identified using the Wilcoxon rank-sum test.

Alpha diversity was assessed using the Chao1 and Shannon indices. Principal component analysis (PCA) was used to explore overall variation in species-level microbial composition. Beta diversity was further evaluated using Bray–Curtis dissimilarity and visualized by principal coordinate analysis (PCoA). Differences in overall microbial community structure between groups were assessed using permutational multivariate analysis of variance (PERMANOVA). Differentially abundant taxa between groups were identified using linear discriminant analysis effect size (LEfSe), with significance defined as a Kruskal–Wallis test *P* < 0.05 and a logarithmic LDA score > 2.0. Differential taxonomic and functional features between groups were identified using the Wilcoxon rank-sum test.

### Untargeted fecal metabolomics analysis

Untargeted fecal metabolomics was performed using LC–MS/MS. Briefly, approximately 100 mg of frozen fecal material was mixed with 800 μL of methanol–water (4:1, v/v) containing internal standards (0.02 mg/mL L-2-chlorophenylalanine and others). Samples were homogenized using a Wonbio-96c frozen tissue grinder (Shanghai Wanbo Biotechnology Co., Ltd.), followed by low-temperature ultrasonication. After incubation at −20 °C for protein precipitation, samples were centrifuged at 13,000 × g for 15 min at 4 °C, and the resulting supernatants were transferred to vials for LC–MS/MS analysis. A pooled quality control (QC) sample was prepared by mixing equal aliquots from all study samples and was injected every five samples throughout the analytical sequence to monitor instrument stability and data reproducibility.

Raw LC–MS files were processed using Progenesis QI (Waters Corporation) for peak alignment, detection, and deconvolution. Metabolite features were annotated by matching MS/MS spectra against the HMDB, METLIN, and Majorbio in-house databases. The resulting data matrix was uploaded to the Majorbio Cloud Platform for preprocessing, including retention of features detected in at least 80% of samples, imputation of missing values using the minimum observed intensity, sum normalization to reduce technical variation, exclusion of QC features with relative standard deviation > 30%, and log10 transformation. After data preprocessing and metabolite annotation, metabolites with annotation confidence at level 2 or higher were retained for descriptive summary and downstream analysis.

Multivariate statistical analyses were performed using the ropls R package. PCA and orthogonal partial least squares discriminant analysis (OPLS-DA) were conducted to evaluate overall metabolic differences between groups. Model robustness was assessed using a permutation test with 200 permutations, in which class labels were randomly reassigned to assess whether the observed model performed better than models generated after random reassignment of class labels. Differential metabolites were screened by integrating multivariate and univariate analyses. Specifically, metabolites with VIP ≥ 1 from the OPLS-DA model, which reflects their contribution to multivariate group discrimination, and *P* < 0.05 from the Wilcoxon rank-sum test were considered differential metabolites. Fold change was calculated to indicate the direction and magnitude of change and was used for volcano plot visualization. Differential metabolites were then mapped to KEGG pathways, and pathway enrichment analysis was performed using the scipy.stats package in Python through the Majorbio Cloud Platform.

### Multi-omics integration and biomarker panel construction

Metagenomic, metabolomic, and clinical data were integrated to explore cross-layer associations in PIMI. Spearman’s rank correlation was used to assess pairwise relationships among key microbial species, key metabolites, and clinical parameters, with *p*-values adjusted using the Benjamini–Hochberg false discovery rate (FDR); significant associations were visualized using correlation heatmaps. Subsequently, key microbial species and key metabolites were jointly incorporated into a Random Forest model to identify the most informative features for discriminating PIMI patients from fertile controls, and top-ranked predictors were selected to construct an integrated microbial-metabolic biomarker panel. Prior to modeling, the input features were standardized using Z-scores. The Random Forest analysis was performed using 500 decision trees, and model performance was evaluated using AUC-based validation across sequential models generated by cumulatively adding top-ranked features. To further assess whether the selected key metabolites and microbial species were independently associated with infertility status, multiple linear regression analyses were performed in GraphPad Prism 8.0 with adjustment for age and smoking status.

## Results

### Baseline characteristics of the study population

A total of 31 participants were included in the present study, consisting of 19 infertile men and 12 fertile controls. Their baseline demographic, lifestyle, and semen characteristics are summarized in Table 1. The infertile group was younger than the control group (*P* = 0.021), whereas body mass index and ethnicity were comparable between the two groups. A significant difference was observed in smoking status (*P* = 0.029), with current or former smokers present only in the infertile group. No significant difference in alcohol consumption was detected. Regarding semen quality, infertile men showed lower sperm concentration (*P* = 0.038) and progressive motility (*P* = 0.003), together with higher round cell counts (*P* = 0.015, Table 1).

**Table 1:** Analysis of the demographics and semen parameters of study participants

Characteristics	Infertile men(19)	Fertile men(12)	*P*-value
Age(year)	31(5.5)	35(2.0)	0.021
Body mass index (kg/m^2^)	24.8(3.7)	23.5(1.7)	0.184
Nation			0.510
Han	17(89.5)	12(100)	
Zang	2(10.5)	0(0)	
Smoking status			0.029
Never smoker	11(57.9)	12(100)	
Ex-smoker	2(10.5)	0(0)	
Current smoker	6(31.6)	0(0)	
Alcohol use			0.252
None	10(52.6)	3(25.0)	
Social	9(47.4)	9(75.0)	
Semen analysis			
Abstinence period(day)	4.1(1.3)	3.8(1.4)	0.469
Semen volume(ml)	4.3(1.7)	3.5(1.2)	0.185
Sperm concentration(million/ml)	47.4(42.6)	78.6(31.9)	0.038
Total sperm count(million)	112(232.2)	258.1(239.6)	0.150
PR(A + B)%	38.0(34.0)	59.5(23.8)	0.003
Round cell(million/ml)	0.4(0.4)	0.2(0.2)	0.015
Forward sperm motility			
VCL (µm/s)	76.6(13.1)	61.0(12.6)	0.004
VSL (µm/s)	32.8(6.9)	28.5(8.3)	0.148
ALH (µm/s)	5.9(1.0)	4.0(0.8)	< 0.001
LIN (%)	43.6(9.7)	50.1(6.8)	0.059
STR (%)	68.6(10.2)	89.1(3.8)	< 0.001
VAP (µm/s)	47.8(6.6)	33.4(7.9)	< 0.001
BCF (times/s)	14.3(2.3)	7.5(0.4)	< 0.001

Data are presented as mean (sd), n (%) or median (IQR)

*PR* Progressive motility, *VCL* Curvilinear Velocity, *VSL* Straight-line Velocity, *ALH* Amplitude of Lateral Head Displacement, *LIN* Linearity, *STR* Straightness Ratio, *VAP* Average Path Velocity, *BCF* Beat Cross Frequency

### Metagenomic profiling reveals alterations in gut microbiome composition and function

**Fig. 2:**
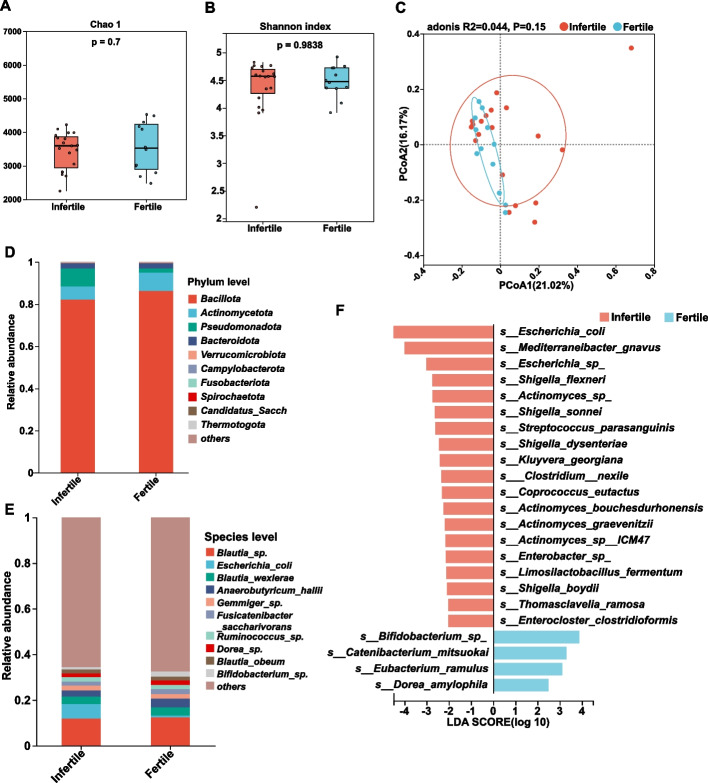
Gut microbial diversity and taxonomic differences between infertile men and fertile controls. **A** Alpha diversity comparison between groups assessed by the Chao1 index. **B** Alpha diversity comparison between groups assessed by the Shannon index. **C** Beta diversity analysis based on Bray–Curtis distances visualized by PCoA; PERMANOVA (adonis) results are shown. **D** Relative abundance of the top 10 taxa at the phylum level. **E** Relative abundance of the top 10 taxa at the species level. **F** LEfSe analysis identifying differentially abundant bacterial species between groups (LDA score > 2.0, *P* < 0.05)

Based on metagenomic sequencing and annotation against the NR database, a total of 8,053 microbial species were identified across all samples and were further analyzed at both taxonomic and functional levels. Gut microbial diversity and overall community structure were first compared between infertile men and fertile controls. Alpha diversity analysis based on the Chao1 and Shannon indices showed no significant differences in microbial richness or diversity between infertile men and fertile controls (Chao1, *P* = 0.7; Shannon, *P* = 0.9838; Fig. 2A, B). Consistently, beta diversity based on Bray–Curtis distances demonstrated no significant separation between groups in PCoA (adonis *R*^2^ = 0.044, *P* = 0.15; Fig. 2C). At the phylum level, the overall microbial composition was broadly similar between groups (Fig. 2D), and no marked global shift was observed at the species level (Fig. 2E). Although no marked global compositional shift was observed, exploratory Wilcoxon rank-sum tests for the taxa displayed in Fig. 2 identified significant differences for *Pseudomonadota* at the phylum level (*P* = 0.0089) and *Escherichia coli* at the species level (*P* = 0.00192). The corresponding results are summarized in Supplementary Table S1.

Nevertheless, Linear Discriminant Analysis Effect Size (LEfSe) analysis identified distinct species-level signatures between infertile men and fertile controls. A total of 23 bacterial species were differentially abundant with an LDA score > 2.0 and *P* < 0.05 (Fig. 2F), suggesting selective microbial alterations in infertile men despite the absence of global community-level differences.

**Fig. 3:**
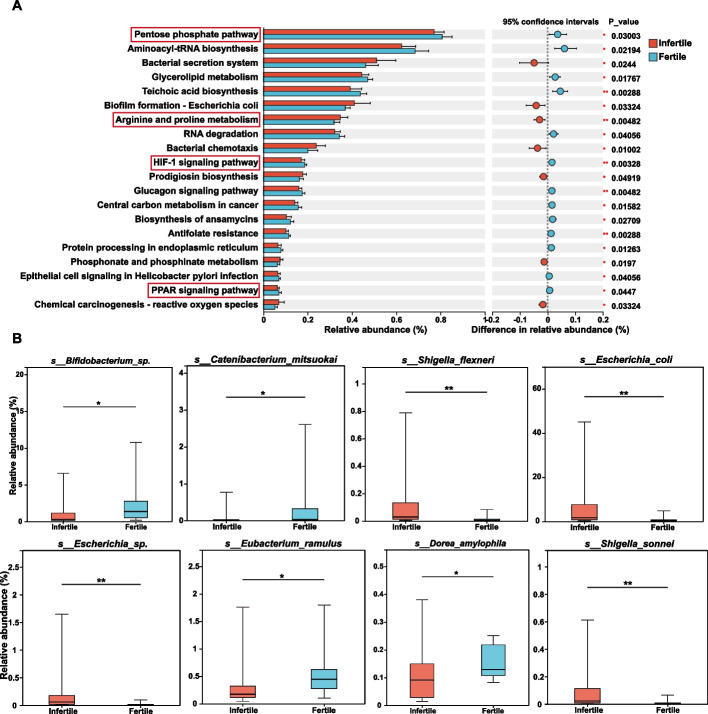
Differential microbial functional pathways and representative species in infertile men and fertile controls. **A** Top 20 differentially abundant KEGG functional pathways between infertile men and fertile controls. The mean relative abundance (%) of each pathway in the two groups, the between-group difference in relative abundance (%), 95% confidence intervals, and corresponding *P* values are shown. Group differences were assessed using the Wilcoxon rank-sum test. **B** Boxplots showing the species-level relative abundance (%) of eight representative microbial species in infertile men and fertile controls. Group differences were assessed using the Wilcoxon rank-sum test

To further characterize functional alterations in the gut microbiome, functional profiling was performed based on metagenomic annotation against the KEGG database. A total of 53 microbial functional pathways were identified as significantly different between infertile men and fertile controls (*P* < 0.05, Supplementary Table S2). The top 20 differentially abundant pathways are presented in Fig. 3A. These pathways were mainly involved in metabolic processes and bacterial functional activities, including the pentose phosphate pathway, aminoacyl-tRNA biosynthesis, and bacterial secretion system, suggesting altered microbial functional potential in infertile men. Among the altered KEGG pathways, we further highlighted four representative pathways, namely the pentose phosphate pathway, arginine and proline metabolism, HIF-1 signaling pathway, and PPAR signaling pathway, because they were significantly altered and were considered biologically relevant to microbial carbohydrate metabolism, amino acid metabolism, and metabolism-associated regulatory processes potentially relevant to male reproductive health.

To clarify the relationship between pathway-level functional alterations and taxonomic features, we further summarized representative genes identified within these four pathways and their related microbial species (Supplementary Table S3). Differential species were initially identified from the significantly altered taxa by LEfSe analysis. However, for pathway-based interpretation, we further prioritized species showing gene-level links to the selected pathways, rather than relying on LEfSe significance alone. Based on this integrative strategy, eight representative microbial species were selected for presentation in Fig. 3B. Among them, *Shigella flexneri, Shigella sonnei, Escherichia coli,* and *Escherichia sp.* were enriched in infertile men, whereas *Bifidobacterium sp., Catenibacterium mitsuokai, Eubacterium ramulus,* and *Dorea amylophila* were more abundant in fertile controls.

### Untargeted metabolomic profiling reveals altered metabolic signatures

**Fig. 4:**
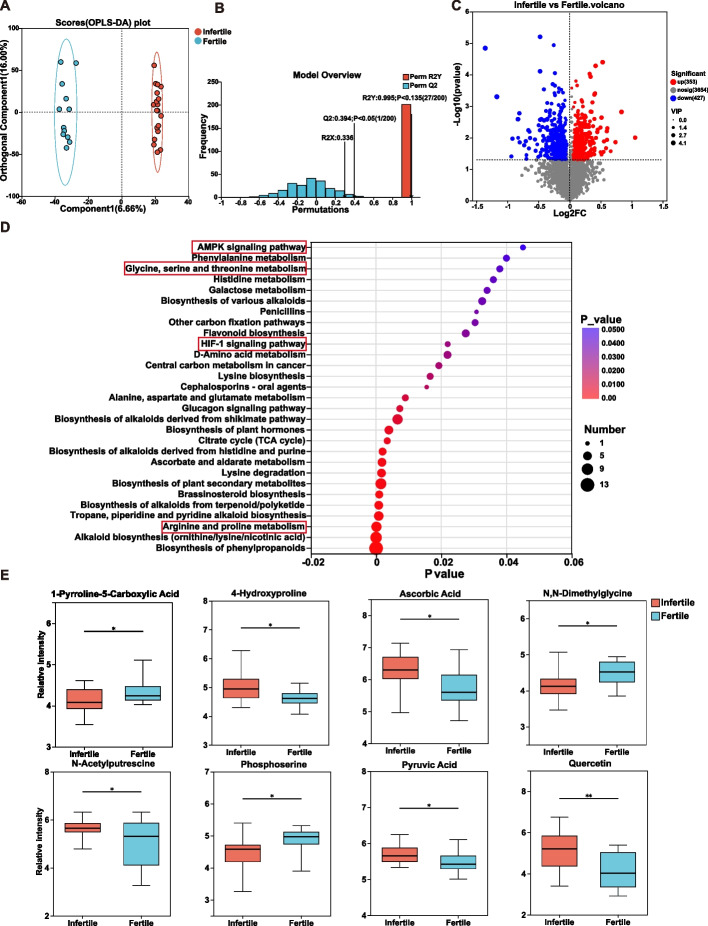
Untargeted fecal metabolomics identifies differential metabolites and enriched metabolic pathways. **A** OPLS-DA score plot showing separation of fecal metabolomic profiles between infertile men and fertile controls. **B** Permutation test for the OPLS-DA model (200 permutations). The distribution of permuted R2Y and Q2 values is shown to evaluate model robustness and the risk of overfitting; the observed model values are indicated for comparison. The red bars represent permuted R2Y values and the blue bars represent permuted Q2 values. **C** Volcano plot showing the distribution of metabolite changes between infertile men and fertile controls according to fold change and *P* value. Red dots indicate metabolites with increased abundance, blue dots indicate metabolites with decreased abundance, and grey dots represent non-significant metabolites (*P* ≥ 0.05). **D** KEGG pathway enrichment analysis of differential metabolites between infertile men and fertile controls. The x-axis shows the enrichment significance (*P* value), dot size represents the number of differential metabolites mapped to each pathway, and dot color indicates the significance level. Pathways with *P* < 0.05 were considered significantly enriched. **E** Boxplots showing the normalized metabolite peak intensity values (displayed as relative intensity) of eight representative metabolites mapped to key enriched pathways in infertile men and fertile controls. The center line indicates the median, the box bounds indicate the upper and lower quartiles (Q3 and Q1), and the whiskers indicate the maximum and minimum values. Group differences were assessed using the Wilcoxon rank-sum test. Significance is indicated as follows: *0.01 < *P* ≤ 0.05, **0.001 < *P* ≤ 0.01, ***P ≤ 0.001

Untargeted fecal metabolomic profiling using LC–MS/MS was performed to compare metabolic profiles between infertile men and fertile controls. After data preprocessing and metabolite annotation, a total of 4,434 metabolites were identified with annotation confidence at level 2 or higher (Supplementary Table S4), including 2,116 metabolites detected in positive ionization mode and 2,318 metabolites detected in negative ionization mode. Unsupervised PCA showed a modest trend toward separation between infertile men and fertile controls, although substantial overlap remained between groups (Fig. S1). When metabolites from both ionization modes were combined for supervised multivariate analysis, OPLS-DA suggested further group discrimination (Fig. 4A). To evaluate model robustness, 200 permutation tests were performed (R2X = 0.336, R2Y = 0.995, Q2 = 0.394; Fig. 4B). These results provide supportive evidence for metabolic differences between groups, but should be interpreted cautiously given the supervised nature of OPLS-DA and the limited sample size. Differential metabolite analysis identified 780 metabolites that differed significantly between infertile men and fertile controls (VIP > 1, *P* < 0.05, Supplementary Table S5), including 353 upregulated and 427 downregulated metabolites in the infertile group. The distribution of differential metabolites is shown in the volcano plot (Fig. 4C). After multiple-testing correction, most of these metabolite-level signals were attenuated and should therefore be interpreted cautiously.

KEGG pathway enrichment analysis of differential metabolites identified 29 enriched pathways (*P* < 0.05), mainly related to amino acid metabolism and metabolic regulation (Fig. 4D). All enriched KEGG pathways and their corresponding mapped differential metabolites are listed in Supplementary Table S6. For downstream biological interpretation, we focused on arginine and proline metabolism, HIF-1 signaling pathway, glycine, serine and threonine metabolism, and AMPK signaling pathway, as these pathways were considered biologically relevant to amino acid metabolism, redox homeostasis, energy metabolism, and male reproductive function, rather than being selected solely according to statistical ranking. Eight significantly altered metabolites mapped to these pathways were further examined, including 1-pyrroline-5-carboxylic acid, 4-hydroxyproline, ascorbic acid, N,N-dimethylglycine, N-acetylputrescine, phosphoserine, pyruvic acid, and quercetin (Fig. 4E).

### Integrative correlation analysis of microbial species, metabolites, and clinical parameters

**Fig. 5:**
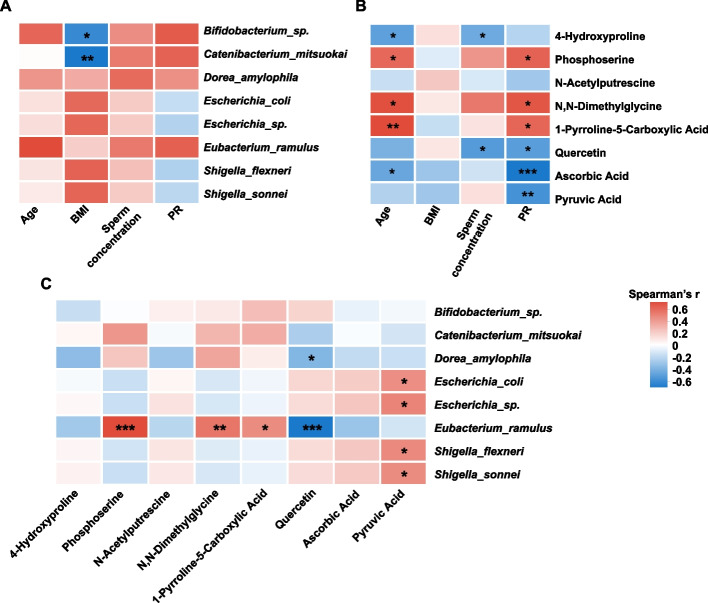
Integrative correlation analysis linking microbial species, metabolites, and clinical parameters. **A** Spearman correlation heatmap showing associations between eight key microbial species and clinical parameters. **B** Spearman correlation heatmap showing associations between eight key metabolites and clinical parameters. **C** Cross-omics Spearman correlation heatmap illustrating associations between key microbial species and key metabolites. Correlation coefficients are shown, and significant associations were determined after FDR correction

Spearman correlation analyses revealed significant associations among key gut microbial species, key metabolites, and clinical parameters. Several microbial species showed notable correlations with clinical traits. In particular, *Bifidobacterium sp.* and *Catenibacterium mitsuokai* were negatively correlated with BMI (Fig. 5A), whereas pathogenic taxa enriched in infertile men (including *Escherichia coli, Escherichia sp., Shigella flexneri*, and *Shigella sonnei*) tended to exhibit opposite correlation patterns with semen quality indicators.

Consistently, metabolite-phenotype correlation analysis showed that multiple key metabolites were significantly associated with clinical parameters (Fig. 5B). Notably, ascorbic acid and pyruvic acid displayed a strong negative correlation with progressive sperm motility (PR), while 1-pyrroline-5-carboxylic acid, phosphoserine, and N,N-dimethylglycine were significantly correlated with age and semen quality indicators.

Furthermore, integrative correlation analysis between microbial species and metabolites showed coordinated cross-omics patterns (Fig. 5C). Multiple taxa enriched in infertile men, including *Escherichia/Shigella* species, were positively correlated with pyruvic acid. In contrast, *Eubacterium ramulus* was positively correlated with 1-pyrroline-5-carboxylic acid, phosphoserine, and N,N-dimethylglycine, but negatively correlated with quercetin. These findings provide descriptive support for coordinated microbe–metabolite patterns in infertile men.

### Predictive modeling based on integrated microbial and metabolic features

**Fig. 6:**
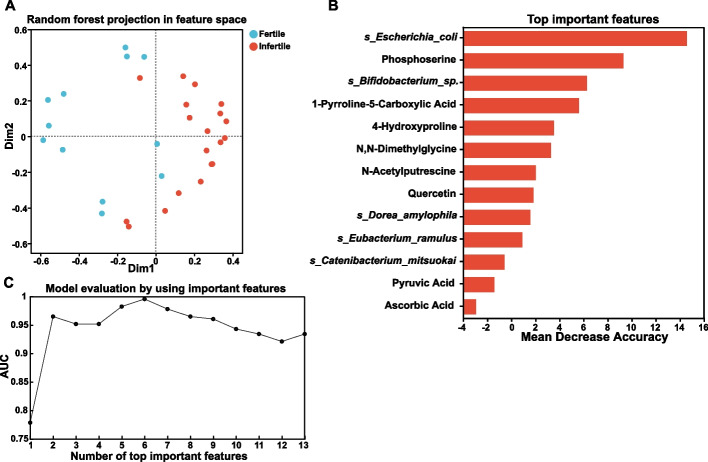
Random forest modeling based on integrated microbial and metabolic features. **A** Sample projection plot from the Random Forest model showing separation between infertile men and fertile controls based on integrated microbial and metabolic features. **B** Top important features ranked by mean decrease in accuracy, highlighting the variables contributing most to classification performance. **C** AUC-based model evaluation according to the number of top-ranked features included in the model

Random forest analysis was performed by integrating eight key microbial species and eight key metabolites. The integrated Random Forest model showed separation between infertile men and fertile controls in the model visualization (Fig. 6A). Variable importance ranking based on mean decrease accuracy indicated that both microbial and metabolic features contributed to classification performance, with *Escherichia coli*, phosphoserine, *Bifidobacterium sp.*, 1-pyrroline-5-carboxylic acid, and 4-hydroxyproline among the top contributors (Fig. 6B). Model performance evaluation further showed that the AUC increased rapidly as the number of top-ranked features increased and reached the highest value when six features were included, followed by a stable plateau with additional features (Fig. 6C), suggesting that the integrated microbial-metabolic panel may have discriminatory potential in this cohort.

To further assess whether these key microbial and metabolic features were independently associated with infertility status, multiple linear regression analyses were performed with adjustment for age and smoking status. Among the eight selected metabolites, six remained significantly associated with infertility status, including phosphoserine, 4-hydroxyproline, N-acetylputrescine, quercetin, ascorbic acid, and pyruvic acid, whereas 1-pyrroline-5-carboxylic acid and N,N-dimethylglycine showed similar but non-significant trends. Similarly, six of the eight selected microbial species remained significantly associated with infertility after adjustment, including lower abundances of *Eubacterium ramulus* and *Dorea amylophila* and higher abundances of *Escherichia coli, Escherichia sp., Shigella flexneri*, and *Shigella sonnei* in infertile men. In contrast, *Bifidobacterium sp.* and *Catenibacterium mitsuokai* showed directionally consistent but non-significant associations (Supplementary Table S7).

## Discussion

This study suggests that primary idiopathic male infertility is characterized by coordinated alterations in gut microbial species, microbial functional potential, and fecal metabolic profiles rather than overt disruption of overall gut microbial diversity. To our knowledge, this is among the first studies to integrate shotgun metagenomics and untargeted fecal metabolomics to characterize gut microbial-metabolic signatures in PIMI. Although alpha and beta diversity measures showed broadly comparable community structure between infertile men and fertile controls, distinct species-level differences were observed, including enrichment of potentially pathogenic taxa such as *Shigella flexneri, Shigella sonnei, and Escherichia coli*, together with reduced abundance of potentially beneficial taxa including *Bifidobacterium sp*. and *Eubacterium ramulus*. At the metabolic level, several altered metabolites were identified, particularly phosphoserine and quercetin, which are related to amino acid metabolism and redox regulation. Cross-omics correlation analysis further suggested coordinated microbe-metabolite patterns, including positive associations between *Escherichia/Shigella*-related species and pyruvic acid, as well as a negative correlation between *Eubacterium ramulus* and quercetin, although these relationships should be interpreted cautiously because the selected microbial species and metabolites were partly prioritized based on related pathway-level findings. In addition, integrated Random Forest modeling suggested that discriminatory performance peaked with a limited set of features, with *Escherichia coli*, phosphoserine, and *Bifidobacterium sp*. ranking among the most informative predictors. Together, these findings suggest that PIMI is accompanied by selective microbial and metabolic perturbations that may be relevant to semen quality.

The gut microbiome is increasingly recognized as a central regulator of host metabolism, immune activity, and endocrine signaling, providing a biological basis for its potential involvement in male reproductive health through the gut-testis axis [7–10]. Although several studies have reported associations between gut microbial dysbiosis and impaired spermatogenesis, existing human evidence has largely focused on taxonomic profiling in heterogeneous infertility phenotypes, with limited integration of microbial functional potential and metabolomic outputs [11, 12]. By integrating shotgun metagenomics and untargeted fecal metabolomics, our study expands current knowledge of gut microbial-metabolic signatures in PIMI.

At the metagenomic level, PIMI was associated with selective taxonomic shifts and altered microbial functional potential. Although overall microbial diversity was broadly comparable between infertile men and fertile controls, species-level differences were evident, with enrichment of potentially pathogenic taxa such as *Shigella flexneri*, *Shigella sonnei, Escherichia coli,* and *Escherichia sp.*, whereas *Bifidobacterium sp.*, *Catenibacterium mitsuokai, Eubacterium ramulus,* and *Dorea amylophila* were relatively more abundant in fertile controls. These findings are in line with prior human studies showing that infertility can be accompanied by discrete compositional shifts without marked changes in global microbial diversity [11, 12].

Importantly, the observed taxonomic alterations were accompanied by functional remodeling of the gut microbiome, with multiple KEGG pathways showing significant differences between groups. We focused on four altered pathways, namely the pentose phosphate pathway, arginine and proline metabolism, HIF-1 signaling pathway, and PPAR signaling pathway, because they were functionally relevant to microbial carbohydrate metabolism, amino acid metabolism, and metabolism-associated regulatory processes potentially related to male reproductive physiology. The species shown in Fig. 3B were initially selected from differentially abundant taxa, whereas the pathway-oriented interpretation presented here mainly emphasizes representative species with gene-level links to these altered pathways. For example, *Bifidobacterium* species are known to utilize carbohydrate metabolism through a modified pentose phosphate pathway and may contribute to NADPH generation and redox homeostasis [19, 20], whereas *Enterobacteriaceae*-related taxa such as *Escherichia* and *Shigella* harbor metabolic functions related to amino acid biosynthesis and arginine metabolism [21, 22]. Given that the pentose phosphate pathway is a major source of NADPH, its alteration may contribute to oxidative stress imbalance, which is closely related to impaired sperm function [23]. Arginine and proline metabolism is linked to polyamine production and nitric oxide regulation, both of which are critical for spermatogenesis and sperm motility [24, 25]. Beyond metabolic rewiring, taxa such as *Eubacterium ramulus* and *Dorea amylophila* may influence the intestinal metabolic environment through microbiome-derived metabolites, including short-chain fatty acids and flavonoid-related metabolic products. This suggests a possible connection between microbial alterations and biological pathways relevant to reproductive physiology, including hypoxia/redox adaptation and lipid metabolic regulation [26–29]. These microbial-derived metabolites have been implicated in shaping cellular responses to hypoxia, oxidative stress, inflammation, and lipid metabolism. In particular, HIF-1 signaling serves as an important mediator of hypoxic and redox adaptation and has been implicated in the maintenance of testicular homeostasis under metabolic challenge [30, 31]. In parallel, PPAR signaling represents an important regulatory axis integrating lipid metabolism and inflammatory tone, with established roles in Sertoli cell function and steroidogenesis in male reproduction [32, 33].

From a metabolic perspective, untargeted fecal metabolomics revealed marked alterations in infertile men, with 780 differential metabolites and enrichment of 29 KEGG pathways. Notably, the enriched pathways were mainly related to amino acid metabolism and metabolic regulation, supporting the presence of metabolic perturbations associated with male infertility [16]. At the same time, because many pathway- and metabolite-level signals were attenuated after multiple-testing correction, these findings should be interpreted cautiously. Among these, four pathways were highlighted based on nominal enrichment significance and biological relevance to processes related to sperm and testicular function, including glycine, serine and threonine metabolism, arginine and proline metabolism, and pathways annotated as AMPK and HIF-1 signaling. For instance, Glycine- and serine-related metabolism contributes to cellular redox buffering and biosynthetic demands during germ cell development [34], whereas arginine metabolism is linked to nitric oxide and polyamine-related processes relevant to spermatogenesis [35]. In parallel, AMPK serves as a key energy sensor in spermatozoa and has been implicated in regulating sperm motility and mitochondrial activity [36], whereas HIF-1 signaling reflects cellular adaptation to hypoxia and oxidative stress and may participate in maintaining testicular homeostasis under metabolic challenge [34, 37]. Consistently, the eight representative metabolites mapped to these pathways showed clear directional shifts between groups. Together, these metabolomic findings suggest potential disturbances in amino acid metabolism, redox balance, and metabolic regulation in PIMI. Importantly, the metagenomic and metabolomic analyses converged on several related biological themes. At the metagenomic level, altered pathways were mainly associated with microbial functional changes related to amino acid metabolism, redox homeostasis, and energy metabolism. Metabolomic enrichment analysis similarly highlighted arginine and proline metabolism, glycine, serine and threonine metabolism, and pathways annotated as AMPK and HIF-1 signaling. Although the specific pathway annotations differed between the two omics layers, both datasets consistently pointed to disturbances in amino acid metabolism, oxidative stress-related processes, and metabolic homeostasis in infertile men.

Beyond pathway-level alterations, integrative correlation analyses revealed coordinated relationships among key microbial species, fecal metabolites, and clinical parameters. In the correlation analysis between clinical parameters and key taxa, *Bifidobacterium sp.* and *Catenibacterium mitsuokai* were both negatively correlated with BMI. A systematic review including 32 cross-sectional studies similarly reported an inverse association between *Bifidobacterium* and BMI/obesity, whereas the association with *Catenibacterium* was inconsistent with our findings [38]. Thus, the relationship between *Catenibacterium mitsuokai* and BMI warrants further validation. Microbe-metabolite correlations also supported the biological coherence of the multi-omics findings. *Enterobacteriaceae*-related taxa enriched in infertile men, including *Escherichia* and *Shigella* species, were positively correlated with pyruvic acid, suggesting a link between microbial shifts and altered energy-related metabolites. Pyruvate represents a central node of microbial carbohydrate fermentation and host-microbe carbon metabolism, and its accumulation may indicate enhanced glycolytic flux and disruption of downstream conversion into short-chain fatty acids [39]. Notably, *Escherichia coli* can exhibit pyruvate accumulation and production under specific metabolic configurations, supporting the biological plausibility of the observed *Escherichia-*pyruvate correlation [40]. In parallel, *Shigella* has been shown to reroute central carbon metabolism and preferentially exploit pyruvate to support rapid growth, providing further support for the *Enterobacteriaceae*-pyruvate association pattern observed in infertile men [41]. In contrast, *Eubacterium ramulus* exhibited a strong negative correlation with quercetin, consistent with prior evidence that this commensal actively degrades dietary flavonoids including quercetin [42]. Taken together, these correlations suggest coordinated relationships among altered microbial species, fecal metabolites, and semen-related clinical parameters in infertile men. In particular, *Escherichia/Shigella*-enriched taxa were positively correlated with pyruvic acid, while pyruvic acid was negatively associated with progressive sperm motility. In contrast, *Eubacterium ramulus*, which was more abundant in fertile controls, showed positive associations with 1-pyrroline-5-carboxylic acid, phosphoserine, and N,N-dimethylglycine, together with a negative association with quercetin, and was also positively related to semen quality indicators. These patterns suggest an overall relationship between microbial alterations, metabolic remodeling, and clinical phenotypes in PIMI.

Notably, integration of microbial and metabolic features enabled construction of a model with discriminatory potential. Using a Random Forest-based integrative strategy, we found that a small set of gut microbial species and metabolites could distinguish infertile men from fertile controls in this cohort. Model evaluation based on AUC within the Random Forest framework suggested that predictive performance improved with the inclusion of top-ranked features and appeared to plateau when approximately five to seven features were incorporated. The features contributing to model performance originated from complementary biological layers, including microbial taxa involved in metabolic processing and metabolites related to amino acid metabolism and redox-associated pathways. Some top-ranked metabolites were also associated with age, indicating that part of the observed discriminatory performance may reflect age-related variation rather than disease-specific signals. After adjustment for age and smoking status, most selected microbial species and metabolites remained associated with infertility status, and the overall direction of these associations was unchanged. However, because both age and smoking are known to influence gut microbial composition and host metabolic homeostasis [43, 44], residual confounding cannot be completely excluded, particularly given the relatively small sample size of the present study. This is especially relevant to the enrichment of *Escherichia/Shigella*-related taxa, which may be partially affected by smoking-related microbial shifts and therefore should be interpreted with caution [45]. The current model should therefore be interpreted as exploratory rather than a definitive predictive signature. This cross-layer integration is broadly consistent with previous studies suggesting that multi-omics-based models may improve disease classification and biomarker discovery [17, 18]. However, further validation in larger, better-balanced cohorts is required before the robustness and biological relevance of this model can be established.

Several limitations should be acknowledged. First, the sample size was relatively small, which may have limited statistical power, increased the multiple-testing burden inherent to multi-omics analyses, and reduced the generalizability of the findings. Therefore, the identified microbial and metabolic signatures should be interpreted cautiously and validated in larger independent cohorts. Second, the cross-sectional design precludes causal inference, and longitudinal or interventional studies are needed to clarify whether gut microbiome alterations contribute to impaired spermatogenesis or occur secondary to host metabolic disturbances. Third, although we performed multivariable adjustment for age and smoking status, residual confounding cannot be completely excluded. This is particularly relevant given the baseline differences between groups and the possibility that some microbial and metabolic features, including *Escherichia/Shigella*-related taxa and certain top-ranked metabolites, may still partly reflect age- or smoking-related variation rather than disease-specific signals. Fourth, dietary intake was not systematically controlled for in this cohort. Because dietary factors can influence fertility-related phenotypes as well as gut microbial composition and fecal metabolic profiles, unmeasured dietary variation may have contributed to part of the observed between-group differences. Fifth, endocrine data were not systematically collected in this cohort, and therefore the relationship between gut microbiome-metabolome alterations and reproductive hormonal regulation could not be directly evaluated. Sixth, an infertile comparison group with a known etiology was not included in the present study. Inclusion of such a group would have helped distinguish features specific to primary idiopathic infertility from those associated with male infertility more broadly. Finally, although multi-omics integration and Random Forest modeling yielded a parsimonious discriminatory panel, the present model was developed as an exploratory analysis and still requires external validation and mechanistic investigation, including targeted metabolomics and host-level functional assays. In addition, nested cross-validation, systematic effect size reporting, and estimation of 95% confidence intervals for AUC were not performed in the present study. Therefore, the discriminatory performance and feature stability of the integrated model should be interpreted cautiously.

## Conclusion

In summary, this study provides integrated metagenomic and metabolomic evidence that primary idiopathic male infertility is associated with alterations in the gut microbiome and fecal metabolome. These changes were mainly related to amino acid metabolism, redox homeostasis, and metabolic regulation, supporting a potential role of the gut-testis axis in PIMI. Multi-omics integration further identified coordinated microbiome-metabolome patterns that distinguished infertile from fertile men, providing a basis for future mechanistic studies and validation in larger independent cohorts.

## Supplementary Information

Supplementary Material 1.

Supplementary Material 2.

Supplementary Material 3.

Supplementary Material 4.

Supplementary Material 5.

Supplementary Material 6.

Supplementary Material 7.

Supplementary Material 8.

## Data Availability

The metagenomic sequencing data generated in this study have been deposited in the NCBI Sequence Read Archive (SRA) BioProject database under accession number PRJNA1423207. The metabolomics data generated in this study have been deposited in the MetaboLights repository under accession number MTBLS13886 and are publicly available at https://www.ebi.ac.uk/metabolights/MTBLS13886.

## Abbreviations

PIMI: Primary idiopathic male infertility
LC–MS/MS: Liquid chromatography-tandem mass spectrometry
KEGG: Kyoto Encyclopedia of Genes and Genomes
NOA: Non-obstructive azoospermia
WHO: World Health Organization
QC: Quality control
PCA: Principal component analysis
OPLS-DA: Orthogonal partial least squares discriminant analysis
VIP: Variable importance in projection
BMI: Body mass index
FDR: False discovery rate
AUC: Area under the curve
ROC: Receiver Operating Characteristic
PCoA: Principal coordinates analysis
LEfSe: Linear Discriminant Analysis Effect Size
LDA: Linear Discriminant Analysis
PPAR: Peroxisome Proliferator-Activated Receptor
AMPK: AMP-Activated Protein Kinase
HIF-1: Hypoxia-Inducible Factor 1
