# Supplementary figures and images for: Functional remodeling of the gut microbiome and metabolome in primary idiopathic male infertility
Source: BMC Microbiol. 2026 Apr 24;26:540. doi: 10.1186/s12866-026-05064-x (PMC13244902; doi:10.1186/s12866-026-05064-x)

**Scores(PCA) plot**

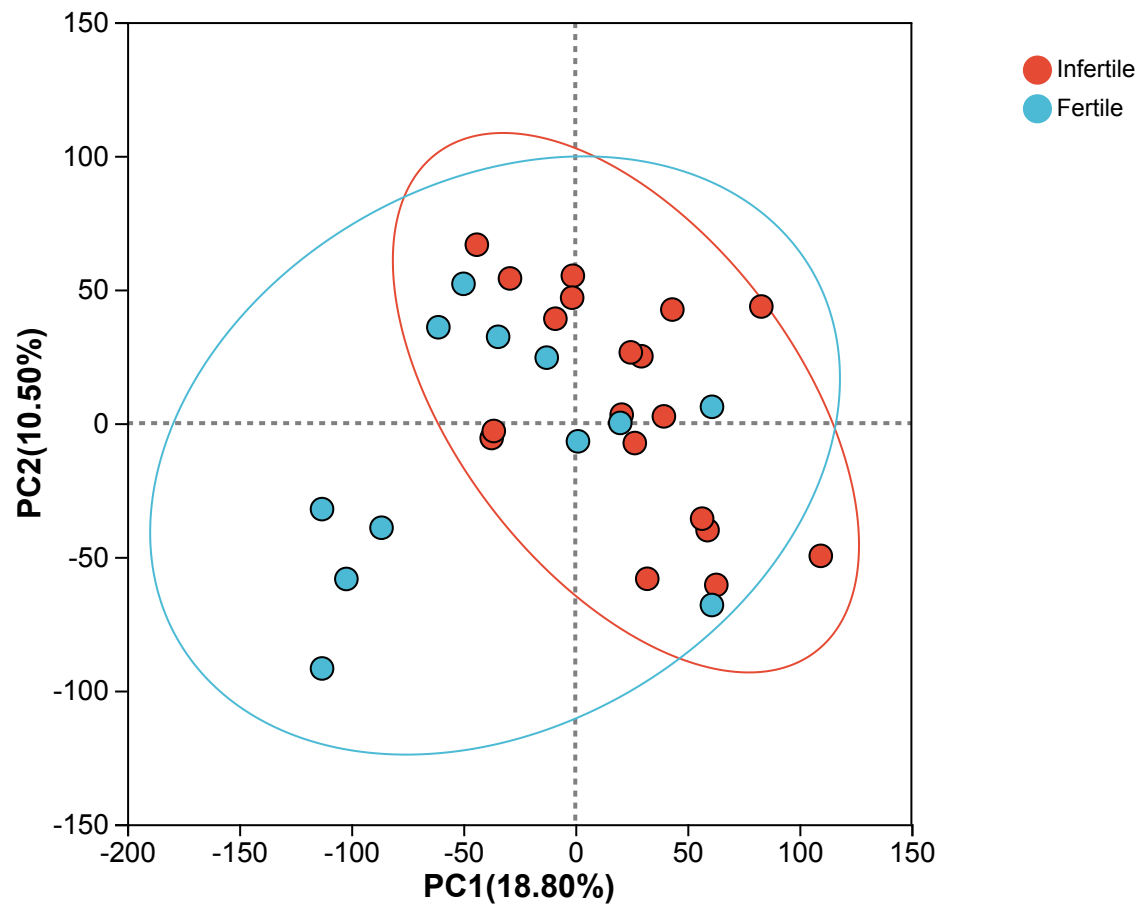

Supplement: Supplementary file 1 — Supplementary Material 1. [file 12866_2026_5064_MOESM1_ESM.pdf]
